# *De Novo* Transcriptome Analysis of Durum Wheat Flag Leaves Provides New Insights Into the Regulatory Response to Elevated CO_2_ and High Temperature

**DOI:** 10.3389/fpls.2019.01605

**Published:** 2019-12-06

**Authors:** Rubén Vicente, Anthony M. Bolger, Rafael Martínez-Carrasco, Pilar Pérez, Elena Gutiérrez, Björn Usadel, Rosa Morcuende

**Affiliations:** ^1^Institute of Natural Resources and Agrobiology of Salamanca (IRNASA), Consejo Superior de Investigaciones Científicas (CSIC), Salamanca, Spain; ^2^Institute for Biology 1, RWTH Aachen University, Aachen, Germany; ^3^Institute of Bio- and Geosciences, IBG-2: Plant Sciences, Forschungszentrum Jülich, Jülich, Germany

**Keywords:** climate change, elevated CO_2_, high temperature, RNA sequencing, transcriptome, durum wheat

## Abstract

Global warming is becoming a significant problem for food security, particularly in the Mediterranean basin. The use of molecular techniques to study gene-level responses to environmental changes in non-model organisms is increasing and may help to improve the mechanistic understanding of durum wheat response to elevated CO_2_ and high temperature. With this purpose, we performed transcriptome RNA sequencing (RNA-Seq) analyses combined with physiological and biochemical studies in the flag leaf of plants grown in field chambers at ear emergence. Enhanced photosynthesis by elevated CO_2_ was accompanied by an increase in biomass and starch and fructan content, and a decrease in N compounds, as chlorophyll, soluble proteins, and Rubisco content, in association with a decline of nitrate reductase and initial and total Rubisco activities. While high temperature led to a decline of chlorophyll, Rubisco activity, and protein content, the glucose content increased and starch decreased. Furthermore, elevated CO_2_ induced several genes involved in mitochondrial electron transport, a few genes for photosynthesis and fructan synthesis, and most of the genes involved in secondary metabolism and gibberellin and jasmonate metabolism, whereas those related to light harvesting, N assimilation, and other hormone pathways were repressed. High temperature repressed genes for C, energy, N, lipid, secondary, and hormone metabolisms. Under the combined increases in atmospheric CO_2_ and temperature, the transcript profile resembled that previously reported for high temperature, although elevated CO_2_ partly alleviated the downregulation of primary and secondary metabolism genes. The results suggest that there was a reprogramming of primary and secondary metabolism under the future climatic scenario, leading to coordinated regulation of C-N metabolism towards C-rich metabolites at elevated CO_2_ and a shift away from C-rich secondary metabolites at high temperature. Several candidate genes differentially expressed were identified, including protein kinases, receptor kinases, and transcription factors.

## Introduction

Wheat is one of the most widely cultivated crop plants along the world and is basic for human nutrition in many areas. Although the hexaploid bread wheat accounts for most of the wheat production, the tetraploid durum wheat is a major crop in the Mediterranean basin, used for the production of traditional staple food. Improving wheat yield capacity is an important goal for future global food security under the challenge of global climate change. As atmospheric [CO_2_] (AC) rises to somewhere in the range from 794 to 1,142 µmol mol^−1^ by the end of the 21st century, the global mean surface temperature is expected to increase, especially in the Mediterranean region, where it will be associated with water stress due to a reduction in rainfall (Habash et al., 2009; IPCC, 2013).

While a DNA array is available for bread wheat, quantitative reverse transcription-PCR (qRT-PCR) has been the most commonly used technique for transcript profiling of wheat grown under future climate scenario (Jauregui et al., 2015; Vicente et al., 2015a; Vicente et al., 2016). The development of next-generation high-throughput RNA sequencing technologies (RNA-Seq) provides new advantages for transcriptome analysis, such as higher sensitivity for genes expressed at extremely low or high level, more detailed gene expression profile, and no limitation by the lack of prior knowledge of the genome (Oshlack et al., 2010). RNA-Seq studies in wheat are rapidly increasing (Duan et al., 2012; Oono et al., 2013; IWGSC, 2014; Kumar et al., 2015; Pingault et al., 2015; Curci et al., 2017) thanks to the reconstruction of the whole transcriptome by using *de novo* assembly of short paired-end (PE) reads. This is particularly interesting for non-model organisms, such as durum wheat, due to the scarcity of sequences available in public databases. On the downside, the transcript data sets generated in RNA-Seq experiments are large and complex (Grabherr et al., 2011), needing bioinformatics knowledge and computation facilities to process the data.

Although plant biomass and yield frequently increase with elevated [CO_2_] (EC) (Long et al., 2004; Aranjuelo et al., 2013; Vicente et al., 2015a), long-term exposure to EC often leads to a downregulation of photosynthetic capacity accompanied by a decline of Rubisco activity and amount (Pérez et al., 2005; del Pozo et al., 2007; Aranjuelo et al., 2011; Vicente et al., 2016). This decline can be accounted for by different mechanisms, including a carbohydrate sink limitation (Ainsworth and Rogers, 2007; Taub and Wang, 2008; Aranjuelo et al., 2011), due to faster CO_2_ assimilation, that can repress photosynthetic genes (Moore et al., 1999; Long et al., 2004), or a lower plant N content caused either by restricted N uptake (del Pozo et al., 2007; Taub and Wang, 2008; Jauregui et al., 2016), the inhibition of N assimilation into proteins (Bloom et al., 2002; Bloom et al., 2010; Vicente et al., 2015a; Vicente et al., 2016), N dilution by accumulation of C-rich compounds (Taub and Wang, 2008), or some other unclear mechanisms (Taub and Wang, 2008; Vicente et al., 2016). In durum wheat grown in field chambers at anthesis, the decline of photosynthetic capacity and N compounds induced by EC is associated with a decrease in transcripts for genes involved in photosynthesis and N assimilation (Vicente et al., 2015a). In bread wheat, the decrease in Rubisco protein content and increase of inhibitors, rather than sugar-mediated gene repression, leads to acclimation to EC even under high N conditions (Pérez et al., 2005). In *Arabidopsis*, EC decreases transpiration, which could be related with the observed depletion of leaf N assimilation and mineral status (Jauregui et al., 2016). In addition, EC leads to an increase in transcripts for genes related to respiration, development, defense, signaling, and sugar content (Leakey et al., 2009; Fukayama et al., 2011; Markelz et al., 2014; Vicente et al., 2015a). In line with these observations, we have reported that EC leads to changes in protein content that enhance C storage and glycolysis (Aranjuelo et al., 2011). Nevertheless, significant disparity exists for the response to EC between plant species, genotypes, development stages, experimental setups, and CO_2_ fumigation methods.

An increase in ambient temperature (AT) will negatively impact on global wheat grain production (Araus et al., 2002; Chauhan et al., 2011) with a predicted decrease of 6% for each °C increase in Earth’s mean temperature (Asseng et al., 2015). Moderate high temperatures (HTs) frequently lead to an increase in photorespiration and an inhibition of photosynthesis (Salvucci and Crafts-Brandner, 2004), promotes early senescence, and is associated with smaller plants (Chauhan et al., 2011). Some studies focused on bread wheat transcript profiling at seedling (Qin et al., 2008; Chauhan et al., 2011) or flowering stage (Kumar et al., 2015) show that most of the heat-responsive genes encode transcription factors (TFs) and proteins involved in transcription, metabolic processes, and stress response, such as hormone, calcium, and sugar signaling, photosynthesis, carbohydrate metabolism, protein modification, and RNA metabolism.

Future enhancement of [CO_2_] can partially compensate the adverse effect of HT (Araus et al., 2002; Habash et al., 2009), especially if other factors, as water stress and/or nutrient deficiencies, are not limiting plant growth and development. In field-grown wheat, Gutiérrez et al. (2009a) pointed that the photochemistry inhibition at HT was counteracted by EC, while Pérez et al. (2011) observed that the negative effect of EC and HT on Rubisco maximum carboxylation activity disappeared for the interaction of both treatments. In agreement with these studies, Chavan et al. (2019) found that EC alleviated heat stress effects on photosynthesis by increasing ribulose bisphosphate regeneration capacity and reducing photochemical damage in a high-yielding wheat cultivar grown in a glasshouse. In a field free air CO_2_ enrichment facility, Fitzgerald et al. (2016) observed that EC stimulated bread wheat biomass and yield and, when combined with HT, buffered the negative effects of heat shocks on grain yield. In contrast, Benlloch-Gonzalez et al. (2014) showed that the positive effect of EC on shoot and root growth was reduced by HT in wheat. Furthermore, the interactive effects of the two factors in wheat negatively affected photosynthetic performance, respiration, N assimilation, and mineral content during grain filling (Jauregui et al., 2015) and repressed genes involved in photosynthesis, respiration, and N metabolism at anthesis (Vicente et al., 2015a). The discrepancies found in wheat responses to EC × HT could likely be explained by the differences in the severity and the duration of the temperature rise, among other factors.

The aim of this work was to further the understanding of the molecular, biochemical, and physiological mechanisms underpinning the response to EC and moderately HT of durum wheat grown in the field in temperature-gradient chambers. The effect of extreme temperature events is out of the scope of this study. Given the importance of these environmental conditions on plant growth and metabolism, here we complement our previous study (Vicente et al., 2015a) to test the hypothesis that, besides primary C-N metabolism, other metabolic processes including cell expansion, hormone synthesis, and secondary metabolism, among others, may be associated with the adaptive mechanisms to these environmental factors, in particular at earlier developmental stages where a lower predominance of resource remobilization is expected as compared to anthesis. The plants were sampled at ear emergence, the start of the period when most of the carbohydrates in the grains are produced by photosynthesis or remobilized (Aranjuelo et al., 2011). We extend precedent qRT-PCR information, limited to genes related to primary C and N metabolism (Vicente et al., 2015a), with transcriptome-wide RNA-Seq analysis combined with functional data to better understand the crop behavior under changing climate conditions. The transcriptome response may improve the understanding of the molecular mechanisms of plant adaptation to EC and HT. Since the transcript changes may not result in parallel alterations in protein activity and metabolites, a complementary study of modifications in metabolites and enzyme activities can provide a more conclusive view.

## Materials and Methods

### Plant Material and Growth Conditions

**Figure 1:**
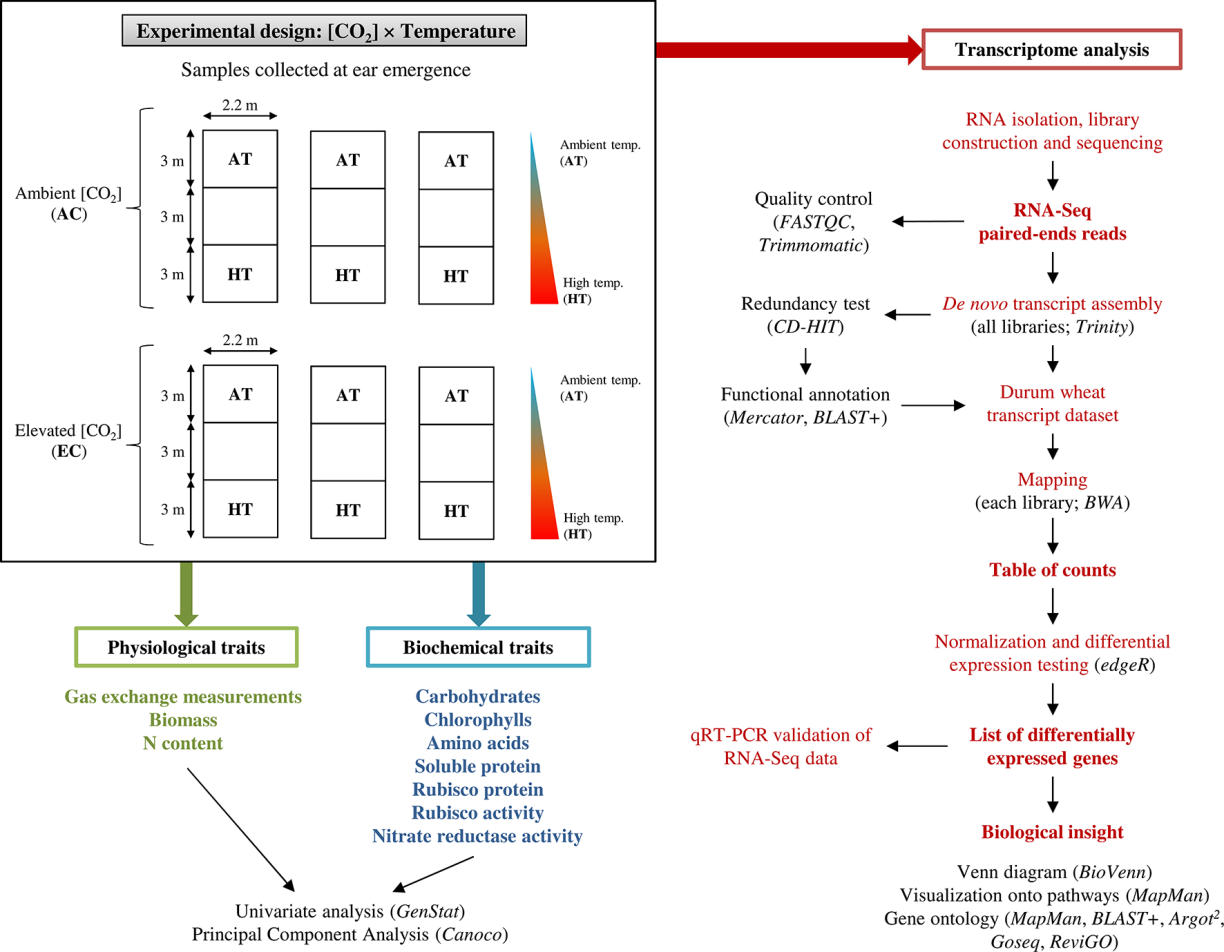
Schematic representation of the experimental setup. Physiological, biochemical, and transcriptome analysis were performed to characterize the durum wheat responses to elevated [CO_2_] and moderate high temperature in durum wheat grown in the temperature-gradient field chambers. RNA sequencing (RNA-Seq) analysis pipeline for the identification of differentially expressed transcripts is shown on right side.

The field experiment was conducted in a clay–sand soil at the experimental fields of the Institute of Natural Resources and Agrobiology of Salamanca (IRNASA-CSIC), in Salamanca, Spain (40°95′ N, 5°5′ W, 800 m a.s.l.), under Mediterranean climate conditions. After adding 60 kg ha^−1^ each of P and K nutrients (as P_2_O_5_ and K_2_O, respectively), durum wheat (*Triticum durum* Desf. cv. Regallo) seeds were sown at a rate of 200 kg ha^−1^ and 0.13 m row spacing on 29 October 2007. We chose cultivar Regallo (released in 1988) because it is a modern semi-dwarf durum wheat genotype with high yield, grain protein content, and adaptability to Mediterranean climate, widely commercialized in our region. N fertilization was applied by hand at 140 kg ha^−1^ as Ca(NO_3_)_2_ on 15 February 2008. This N supply was considered optimal based on previous experience from other experiments in the same location and soil. The crop was watered twice, sometimes once a week with a drip irrigation system, providing an amount of water nearly equivalent to the mean crop evapotranspiration [Allen et al., 2006; values for reference evapotranspiration (ET_o_) and crop coefficient (K_c_) of 3 mm day^−1^ and 1.15, respectively] (**Supplementary Figure 1**). Six temperature-gradient chambers (Pérez et al., 2005; Gutiérrez et al., 2009a; Gutiérrez et al., 2009b; Gutiérrez et al., 2013; Vicente et al., 2015a) were mounted over the crop on 27 February 2008. They were 9 m long, 2.2 m wide, and 1.7 m high with transparent walls and roof, divided into three modules separated by polycarbonate septa to reduce the mixing of air between modules due to convection. Three of them were kept at ambient [CO_2_], 370 µmol mol^−1^, while [CO_2_] was increased to 700 µmol mol^−1^ in other three by injecting pure CO_2_ at the two inlet fans of each chamber during the light hours, controlled by an infrared gas analyzer as described in Pérez et al. (2005) and Gutiérrez et al. (2009b). The temperatures at the extreme chamber modules were set at ambient and 4 °C warmer by using two inlet and one outlet fans at the required speed, in combination with fan heaters to create a temperature gradient between modules (Pérez et al., 2005; Gutiérrez et al., 2009b) (**Supplementary Figure 1**). Plants received a total of 255 mm from sowing to ear emergence (**Supplementary Figure 1**). Flag leaves were harvested on 7 May 2008 at ear emergence (Zadoks 59) and 2 days after this stage, respectively, in plants grown at ambient and high temperature; for simplicity, thereafter this stage will be referred to as ear emergence. The leaves were immediately frozen in liquid N and stored at −80°C for biochemical and molecular analyses. In summary, four treatments were considered in this study for further analysis: ambient CO_2_ and temperature (AC-AT), ambient CO_2_ and high temperature (AC-HT), elevated CO_2_ and ambient temperature (EC-AT), and elevated CO_2_ and high temperature (EC-HT). A schematic representation of the experimental design is detailed in **Figure 1**.

### Physiological and Biochemical Parameters

Gas exchange in the central segment of the flag leaves was recorded between 3 and 8 h after the start of the photoperiod at the respective growth CO_2_ concentration. Photosynthesis rate (A_n_), stomatal conductance (g_s_), transpiration (E), and intercellular CO_2_ concentration (C_i_) were measured at 300 ml min^−1^ air flow rate, 1,500 µmol m^−2^ s^−1^ irradiance, and 25°C temperature using the Peltier system in a 1.7 cm^2^ window leaf chamber connected to a portable infrared gas analyzer CIRAS-2 (PP Systems, UK). The measurements were done when inlet [CO_2_] was stable and after checking for leaks in the chamber. Shoots were collected to determine the dry weight of flag leaves and total shoot after drying in an oven at 60°C for 48 h. N concentration as a percentage of dry matter and the N content in flag leaves and shoots were determined as described by Vicente et al. (2015b). Briefly, dried samples were subjected to Kjeldhal digestion with H_2_SO_4_ using a Se catalyst and adjusting the pH to 3–4 using 1 M triethanolamine buffer (pH 7.2) and 5 M KOH. N, in the form of NH_4_^+^, was spectrophotometrically determined at 340 nm as the NADPH reduced during the conversion of NH_4_^+^ and 2-oxoglutarate into glutamate catalyzed by glutamate dehydrogenase (Ammonia Rapid kit, Megazyme, Ireland). After ethanol/water extraction, soluble sugars (glucose, fructose, sucrose, and fructans) and starch content were measured as mentioned in Morcuende et al. (2004). Firstly, glucose and fructose were measured spectrophotometrically in the supernatant with an assay coupled to NADP reduction based on Jones et al. (1977). After incubating another aliquot with sucrase to metabolize the sucrose in the sample, the released glucose and fructose related with sucrose were determined. Later, in a third aliquot the content of fructans in the form of glucose and fructose were measured after their hydrolysis with fructanases. Finally, starch was measured in the insoluble residue from the extraction after incubation with amyloglucosidase and α-amylase at 37°C overnight. Then, starch was measured in the form of glucose as mentioned above. Chlorophyll (chl) was extracted in 80% acetone and assayed at 645 and 663 nm for the calculation of total chl according to Arnon (1949). Total amino acids content was measured in the ethanol/water extracts by the ninhydrin method (Hare, 1977) as described by Aranjuelo et al. (2011). Soluble proteins were extracted and determined spectrophotometrically according to Bradford (1976). Rubisco protein was separated by SDS-PAGE electrophoresis and the content determined by densitometry (Pérez et al., 2011). Initial and total Rubisco activities were measured using a cascade reaction involving several enzymes exactly as described in Pérez et al. (2011), where activity was at a stoichiometry of 1:2 equivalent to the NADH oxidation recorded at 340 nm. Rubisco activation state was calculated as the ratio between initial and total Rubisco activity. Maximal activity and activation state of nitrate reductase (NR) were assessed by the formation of NO_2_^−^ in the absence or presence of 10 mM Mg^2+^ as described by Morcuende et al. (2004) based on the procedure described by Scheible et al. (1997).

### RNA Isolation, cDNA Library Construction, Illumina Sequencing, and Quality Control Processing of Raw Data

Total RNA was prepared by grinding the flag leaves in liquid N and extracting RNA using the method described by Morcuende et al. (1998). Three biological replicates were used per treatment. RNA integrity/degradation was examined on 1.5% agarose gels (**Supplementary Figure 2**). RNA quality and concentration were assessed using NanoDrop ND-1000 spectrophotometer (Thermo Fisher Scientific, USA) and 2100 Bioanalyzer (Agilent Technologies, Santa Clara, CA) as shown in **Supplementary Table 1**. RIN^e^ (RNA integrity number equivalent) scores were greater than 8.0 for all samples, as recommended by the manufacturer. Twelve cDNA libraries (one per sample) were generated by using Illumina TruSeq RNA Library Preparation Kit (Beckman Coulter Genomics, Beverly, USA). RNA sequencing was performed on each library to generate PE reads of length 100 nucleotides on two lanes of Illumina HiSeq 2000 (Beckman Coulter Genomics, Beverly, USA). Quality of raw data was verified by FastQC software (http://www.bioinformatics.babraham.ac.uk/projects/fastqc/). Adapters and contaminated sequences were removed, and low-quality bases from both ends of each read were trimmed at a Phred quality scores threshold of Q20 (< 1 error per 100 bp) from the raw data in FastQ format using PE mode of Trimmomatic (Bolger et al., 2014). **Figure 1** also contains an overview of the RNA-Seq analysis pipeline followed. Whole data set has been deposited in the European Nucleotide Archive with accession number PRJEB34302.

### *De Novo* Assembly and Functional Annotation

All the quality-controlled PE reads from the 12 libraries were *de novo* assembled using Trinity (Grabherr et al., 2011), with a fixed *k*-mer size of 25, to generate a durum wheat transcript data set. Trinity has a good performance in wheat RNA-Seq experiments (Duan et al., 2012; Oono et al., 2013), and it was the most successful assembler after testing four different ones (Li et al., 2013). With a minimum *k*-mer coverage of 10 (minimum count of *k*-mers to be assembled), 196,843 contigs in 60,209 transcripts (unigenes/loci) were obtained. Transcript redundancy was tested using CD-HIT software with identity of 95%, although the number of redundant transcripts was negligible. Functional annotation of the durum wheat transcript data set was based on the large amount of public data for wheat and other plant species. Firstly, all transcripts were submitted to Mercator web tool (Lohse et al., 2014), which generated functional predictions by searching a variety of reference databases (TAIR release 10; SwissProt/UniProt Plant Proteins; TIGR5 rice proteins; clusters of orthologous eukaryotic genes database, KOG; and conserved domain database, CDD), using a BLAST cutoff of 40. Furthermore, a BLASTN/BLASTX search for all the transcripts against different databases (TAGI release 12 for bread wheat, TIGR release 1 and 2 for bread and durum wheat, *Brachypodium* genome v1.2 at PlantGDB, and rice genome release 7) was achieved using BLAST+ and an E-value cutoff of 1e^−3^ to improve transcript annotation.

### Mapping of Reads to Durum Wheat Transcript Data Set, Normalization, and Identification of Differentially Expressed Transcripts

Quality-controlled PE reads for each sample were mapped back to the durum wheat transcript data set with the alignment program BWA (Li and Durbin, 2009), with the aim to find the location where each short read best matches the reference (Oshlack et al., 2010). The data were summarized in a table of counts, rows for transcripts, and columns for counts, which represent the total number of reads aligning to each non-redundant contig of the durum wheat transcript data set. Differential gene expression analysis across experimental conditions was performed using the R/Bioconductor package edgeR. The library sizes were computed from the column sums of the counts. Using the plants grown under ambient CO_2_ and temperature as the control treatment, we focused on the EC-, HT- and EC × HT-responsive transcripts. We considered as differentially expressed (DE) transcripts the ones with a false discovery rate (FDR) < 0.05 and an induction or repression ratio ≥ two-fold.

### Visualization of RNA-Seq Results and Functional Enrichment Analysis

Venn diagram analysis of DE transcripts was made in BioVenn (Hulsen et al., 2008). Visualization of metabolic pathways was performed using MapMan (Thimm et al., 2004). Previously, MapMan BINs (hierarchical functional categories assigned to certain biological processes) were assigned with Mercator to all input sequences with the aim to create a mapping file for MapMan (see section *De Novo Assembly and Functional Annotation*). For the transcripts incorporated to BIN 35.2 (unknown sequence) by Mercator, but annotated by BLAST+ using TAGI database, *Brachypodium*, and rice genomes, new BINs were assigned. A special BIN category (2.1.3) was created for the genes involved in fructan biosynthesis, due to its strong over-expression under EC in this study and in previous ones of our group (Vicente et al., 2015a; Vicente et al., 2016). Analysis of functional categories of the DE transcripts was performed based on the MapMan categories using the BINs assigned previously. Likewise, Gene Ontology (GO) terms provided by the GO project (http://www.geneontology.org/) were assigned to the DE transcripts based on the GO terms downloaded from the *Brachypodium distachyon* and rice genome annotation projects and UniProtKB database. GO terms for UniProtKB database were assigned after running Argot^2^ tool using default parameters (Falda et al., 2012). The R/Bioconductor package GOseq was used to correct gene length bias. Only GO terms with corrected *P* values <0.05 were considered significantly enriched, and the results were visualized and summarized using a clustering algorithm with the web server ReviGO (Supek et al., 2011) with default parameters.

### Validation of RNA-Seq Data by qRT-PCR

The validation of the RNA-Seq results and reverse transcription reactions were performed using qRT-PCR as described in Vicente et al. (2015a). Two technical replicates were analyzed for each one of the three biological replicates per treatment. The relative transcription levels were analyzed using the 2^−ΔΔCt^ method and compared with expression levels of RNA-Seq. The ADP-ribosylation factor was used as reference gene for qRT-PCR normalization (Vicente et al., 2015a; Vicente et al., 2016) and is listed in **Supplementary Table 2** together with the genes used for the validation.

### Statistical Analysis

The experiment had three blocks with all the factorial combinations of two atmospheric [CO_2_] (370 and 700 µmol mol^−1^) and two temperatures (ambient and 4°C warmer). Treatment effects and interactions were determined through an analysis of variance (ANOVA) using GenStat software for physiological and biochemical parameters. When differences between treatments were significant (*P <*0.05), they were evaluated using the least significant difference test (*LSD*). As mentioned above, DE transcripts were considered significant with FDR <0.05 and a fold-change cutoff of 2 using edgeR. Multivariate statistical analysis of the physiological and biochemical data was performed using principal component analysis (PCA) with Canoco for Windows (Biometris, Plant Research International).

## Results

### Physiological and Biochemical Responses to Elevated CO_2_ and High Temperature

EC stimulated net photosynthetic rates (A_n_) by 32% compared to AC at ear emergence (**Table 1**). This increase was greater in plants grown at AT than HT, although the CO_2_ × temperature interaction did not reach statistical significance. EC led to a reduction of stomatal conductance (g_s_) and transpiration (E), and an increase in intercellular [CO_2_] (C_i_), with greater C_i_ values at AT than HT. Shoot biomass was promoted by 17% by EC, while no differences in leaf dry weight were observed with an elevation of [CO_2_] or temperature. The content of some carbohydrates was altered by EC, HT, or their interaction. Starch content increased by two-fold and glucose decreased by 25% under EC. In contrast, starch decreased by 70% and glucose increased by 60% at HT compared to AT. Furthermore, EC increased significantly fructan content, with larger content at AT relative to HT. Neither fructose nor sucrose contents were modified by growth conditions.

**Table 1:** Photosynthesis rate (A_n_), stomatal conductance (g_s_), transpiration (E), intercellular CO_2_ concentration (C_i_), dry weight (DW), N concentration, total N content (N_t_), chlorophyll (chl), glucose, fructose, sucrose, fructan, starch, total amino acid, soluble and Rubisco protein contents, Rubisco as a percentage of soluble protein, initial and total Rubisco activities (Rbco act.), Rubisco activation state (Rbco %act.), maximal nitrate reductase (NR) activity, and activation (NR %act.) in durum wheat grown at ambient (AC, 370 µmol mol^−1^) or elevated (EC, 700 µmol mol^−1^) [CO_2_] and ambient temperature (AT) or ambient + 4°C (HT).

		CO_2_		Temperature		CO_2_ × temperature			
Parameter	Units	AC	EC	AT	HT	AC-AT	AC-HT	EC-AT	EC-HT
**A_n_**	µmol m^−2^ s^−1^	**24.7 a**	**32.7 b**	29.9	27.5	25.3	24.1	34.6	30.9
**g_s_**	mmol m^−2^ s^−1^	**465 b**	**292 a**	371	386	419	511	323	262
**E**	mmol m^−2^ s^−1^	**4.97 b**	**3.96 a**	4.31	4.62	4.56	5.38	4.06	3.85
**C_i_**	µmol mol^−1^	194	363	301	257	**183 a**	**204 a**	**417 c**	**308 b**
**Leaf DW**	g	0.131	0.141	0.141	0.131	0.130	0.131	0.152	0.130
**Shoot DW**	g	**2.40 a**	**2.80 b**	2.54	2.66	2.40	2.39	2.68	2.92
**Glucose**	µmol g FW^−1^	**8.04 b**	**6.03 a**	**5.42 a**	**8.65 b**	5.96	10.13	4.88	7.17
**Fructose**	µmol g FW^−1^	9.16	10.38	10.05	9.49	8.50	9.82	11.60	9.16
**Sucrose**	µmol g FW^−1^	16.5	23.8	21.8	18.5	16.7	16.4	26.9	20.7
**Fructans**	µmol g FW^−1^	19.0	135.3	95.9	58.5	**33.4 a**	**4.6 a**	**158.4 c**	**112.3 b**
**Starch**	µmol g FW^−1^	**1.54 a**	**3.08 b**	**3.55 b**	**1.07 a**	2.24	0.84	4.86	1.31
**Leaf %N**	% dry weight	**3.89 b**	**3.26 a**	3.73	3.42	4.03	3.75	3.43	3.09
**Shoot %N**	% dry weight	**1.43 b**	**1.13 a**	1.34	1.22	1.52	1.33	1.16	1.10
**Leaf N_t_**	mg N per organ	5.09	4.79	5.26	4.62	5.27	4.90	5.24	4.33
**Shoot N_t_**	mg N per organ	**35.8 b**	29.7 a	33.1	32.4	36.2	35.5	30.0	29.4
**Chl**	mg g FW^−1^	2.91	2.62	2.90	2.63	**3.27 b**	**2.56 a**	**2.53 a**	**2.71 a**
**Amino acids**	µmol g FW^−1^	**20.1 b**	14.6 a	17.1	17.6	21.0	19.3	13.2	16.0
**Soluble protein**	mg g FW^−1^	**33.6 b**	22.9 a	29.6	26.9	36.5	30.8	22.7	23.1
**Rbco protein**	mg g FW^−1^	**19.7 b**	12.6 a	**17.1 b**	**15.3 a**	21.2	18.2	13.0	12.3
**Rbco % sol. protein**	%	**58.5 b**	51.4 a	55.1	54.7	58.7	58.2	51.6	51.3
**Initial Rbco act**.	µmol m^−2^ s^−1^	43.4	29.4	42.9	29.8	**56.1 b**	**30.6 a**	**29.7 a**	**29.0 a**
**Total Rbco act**.	µmol m^−2^ s^−1^	86.0	61.6	84.2	63.3	**105.1 b**	**66.9 a**	**63.4 a**	**59.7 a**
**Rbco %act**.	%	48.4	48.9	51.5	45.8	56.0	40.8	47.0	50.8
**NR activity**	µmol g^−1^ h^−1^	**8.79 b**	**5.43 a**	7.81	6.41	9.76	7.83	5.87	4.99
**NR %act**.	%	57.5	80.3	79.6	58.2	**62.2 a**	**52.8 a**	**97.0 b**	**63.6 a**

Unless otherwise specified, the measurements were carried out on the flag leaf. Each main factor level is the mean of 12 replicates, and factor combinations are means of 6 replicates (half for gas exchange parameters). For each comparison of means, values with different letter are significantly different (P < 0.05) and marked in bold. No letters are added for the CO_2_ × temperature combinations when the interaction is not significant or for the factor main effects when the interaction is significant.

N concentration in leaves and shoots was consistently reduced by EC (16% and 21%, respectively), together with a decline of 17% in total shoot N content (**Table 1**). N-rich compounds were also affected by environmental conditions. Chl content was reduced by EC, HT, or the EC × HT interaction compared to AC and AT. Growth at EC caused a decrease in total soluble proteins (32%) and Rubisco content (36%), together with a decline (12%) of Rubisco as a percentage of soluble protein. HT also decreased significantly Rubisco content by 11%. Enzyme assays for key proteins of C (Rubisco) and N (NR) metabolism also revealed some changes caused by growth conditions. Similar to chl content, initial and total Rubisco activities were remarkably reduced under EC, HT (45–48%), or the EC × HT interaction (36–43%; **Table 1**). NR activity was diminished by 38% under EC, while the activation state of the enzyme strongly increased by 56% compared to AC. This increase in the activation state under EC was not supported at HT.

**Figure 2:**
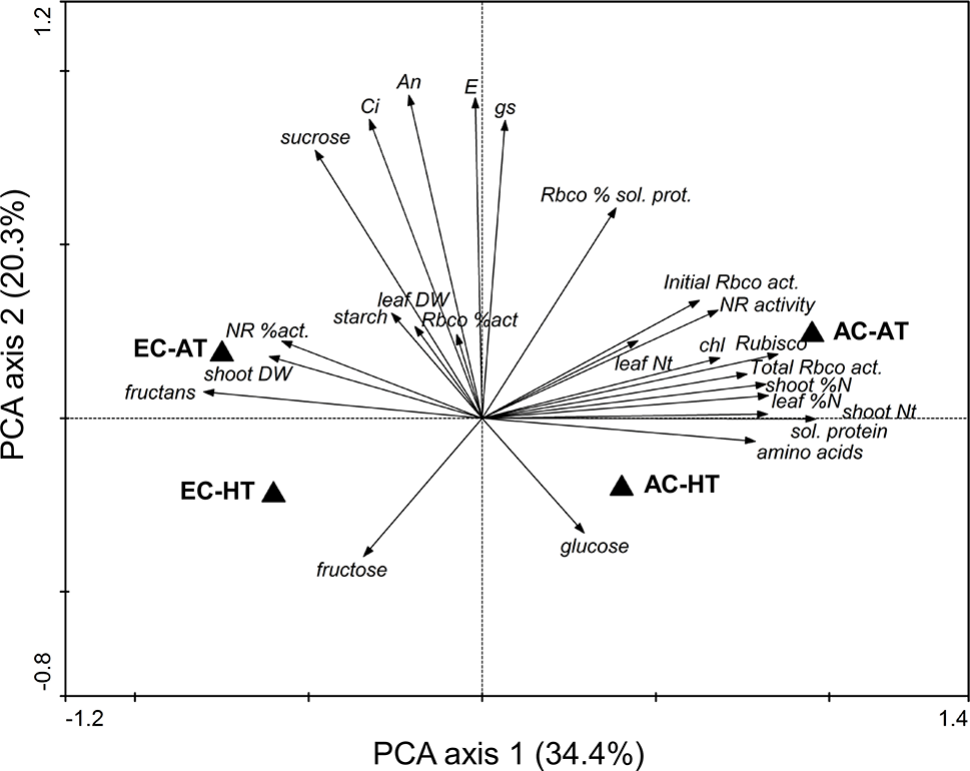
Principal component analysis of the physiological and biochemical traits of durum wheat flag leaves in response to ambient (AC, 370 µmol mol^−1^) or elevated (EC, 700 µmol mol^−1^) [CO_2_] and ambient temperature (AT) or ambient + 4°C (HT). Data analysis was carried out with the results included in **Table 1**.

The differences in physiological and biochemical traits between treatments were supported by PCA (**Figure 2**). There were variations caused by changes in air [CO_2_] and temperature, represented by the first and second principal components, respectively, which were large for air [CO_2_]. Most of the traits related to N content, N-rich compounds, and enzyme activities were concentrated in the same area, showing that the AC-AT control treatment reached the highest values. Warmer temperatures consistently decreased these traits and showed an increase in glucose content. Treatments under EC were clearly separated from those under AC, although differences due to changes in temperature were significantly reduced under EC relative to AC. Carbohydrates tended to increase under EC, especially fructans, starch, and sucrose at AT and fructose at HT. Growth at EC and AT promoted shoot biomass and the activation of NR enzyme.

### Transcriptome Sequencing and *De Novo* Assembly

A total number of 395 million PE short-read sequences (77.2 Gb), were generated for the 12 libraries and used for downstream analysis, with an average of 65.9 million reads per sample (**Supplementary Table 3**). All reads were *de novo* assembled. An average of 88.21% reads was uniquely mapped per library (**Supplementary Table 3**). We obtained a durum wheat transcript data set of 60,209 transcripts. Functional annotation of this transcript data set was achieved by using Mercator tool (15,402 transcripts; 25.6% of the total), durum (4,994 transcripts; 8.3%), and bread (30,713 transcripts; 51.0%) wheat sequences available in TIGR, bread wheat sequences available in TAGI (24,558 transcripts; 40.8%), *Brachypodium* genome (protein sequences; 25,662 transcripts; 42.6%), and rice genome (protein sequences; 25,336 transcripts; 42.1%). Thus 38,869 transcripts were annotated by at least one database. Nonetheless, it is important to consider that some matches described a homologous locus/gene in other species without any annotation. The transcript levels of seven genes involved in primary metabolism were evaluated by qRT-PCR (**Supplementary Table 2**). The results of the relative expression of these genes under EC, HT, and its combination from qRT-PCR and RNA-Seq analysis were compared (**Supplementary Figure 3**). A similar pattern of changes were obtained from both techniques, which validates the RNA-Seq results.

### Identification of Differentially Expressed Transcripts and Their Classification Using MapMan Categories

**Figure 3:**
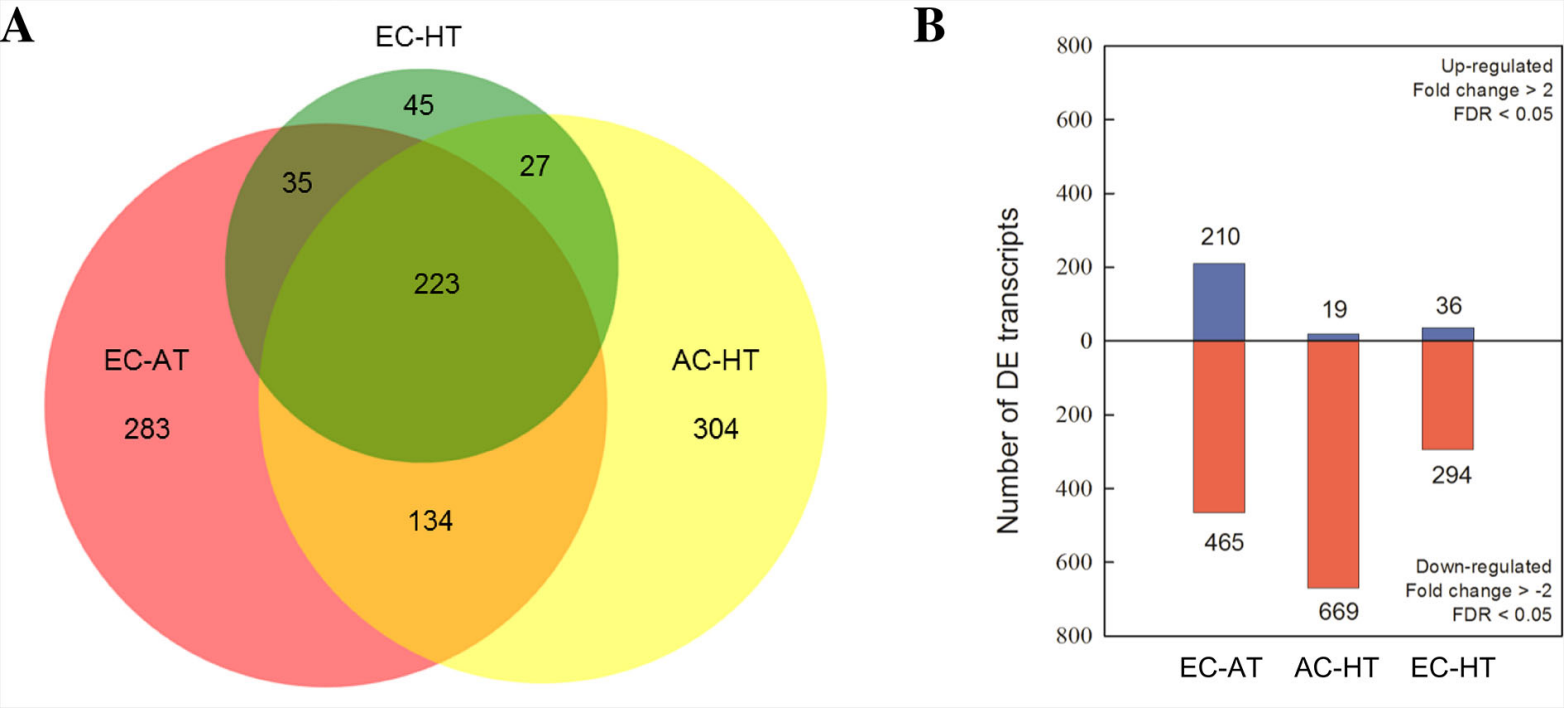
**(A)** Venn diagram analysis of the differentially expressed (DE) transcripts and **(B)** number of DE transcripts which were up (blue) or downregulated (red) under ambient [CO_2_] and high temperature (AC-HT), elevated [CO_2_] and ambient temperature (EC-AT), or elevated [CO_2_] and high temperature (EC-HT), relative to control treatment at ambient [CO_2_] and temperature in durum wheat flag leaves.

Based on FDR values, a total of 1,051 DE transcripts were detected in at least one treatment compared with the control treatment with AC and AT (**Figure 3A**). Venn diagrams exhibited an overlap of 419 DE transcripts between, at least, two treatments, while 223 of them were altered in all three treatment combinations relative to the control. A total of 27% and 29% of the DE transcripts were uniquely responsive to EC and HT, respectively, while only 4% was unique for EC × HT. Upregulation of 210, 19, and 36 genes, and downregulation of 465, 669, and 294 genes was detected under EC, HT, and EC × HT relative to control treatment, respectively (**Figure 3B**). A detailed summary of the DE transcripts is provided in **Supplementary Table 4**.

For the whole durum wheat transcriptome, we annotated 21,512 transcripts (35.7%) with MapMan BINs of known function, after running the Mercator web tool and using the BINs previously assigned to TAGI database and *Brachypodium* and rice genomes. Using these BINs, the DE transcripts were classified into 34 categories (**Table 2**), while 307, 319, and 146 transcripts differentially expressed, respectively, with EC, HT, and EC × HT were assigned to BIN 35 “no ontology/unknown”. The results using the MapMan categories highlight that only a small proportion of genes related to primary and secondary metabolisms were up or downregulated (**Table 2**). Most of the genes that were DE belonged to categories such as stress, RNA and DNA metabolisms, miscellaneous enzyme families, protein and signaling. While several genes were upregulated under EC or the interaction EC × HT relative to control treatment, most of them were downregulated under HT.

**Table 2:** Analysis of the differentially expressed (DE) transcripts based on the functional MapMan categories.

Bin code	Category	Elevated [CO_2_] (EC-AT)			High temperature (AC-HT)			Elevated [CO_2_] x high temperature (EC-HT)		
		Up	Down	Total	Up	Down	Total	Up	Down	Total
1	Photosynthesis	3	7	10	2	6	8	0	6	6
2	Major CHO metabolism	2	0	2	0	0	0	1	0	1
3	Minor CHO metabolism	1	3	4	0	4	4	1	3	4
4	Glycolysis	0	2	2	0	3	3	0	2	2
8	TCA cycle	0	0	0	0	1	1	0	0	0
9	Mitochondrial electron transport	5	1	6	0	1	1	0	1	1
10	Cell wall	1	2	3	0	6	6	1	0	1
11	Lipid metabolism	2	2	4	0	7	7	1	1	2
12	N metabolism	0	1	1	0	1	1	0	0	0
13	Amino acid metabolism	2	5	7	0	6	6	0	4	4
16	Secondary metabolism	11	5	16	0	15	15	1	3	4
17	Hormone metabolism	3	6	9	0	8	8	0	2	2
18	Co-factor and vitamin metabolism	0	0	0	0	1	1	0	0	0
19	Tetrapyrrole synthesis	1	0	1	0	0	0	0	0	0
20	Stress	12	77	89	0	81	81	0	44	44
21	Redox	1	1	2	0	3	3	0	0	0
23	Nucleotide metabolism	1	1	2	0	0	0	0	1	1
24	Biodegradation of xenobiotics	3	0	3	0	0	0	0	0	0
25	C1 metabolism	1	0	1	0	0	0	0	0	0
26	Miscellaneous enzyme families	10	15	25	1	28	29	5	7	12
27	RNA	16	16	32	0	29	29	5	12	17
28	DNA	4	12	16	2	17	19	3	9	12
29	Protein	18	28	46	1	50	51	3	19	22
30	Signaling	3	53	56	2	68	70	1	35	36
31	Cell	5	3	8	0	3	3	0	1	1
33	Development	5	1	6	1	7	8	1	1	2
34	Transport	11	6	17	0	15	15	3	7	10
35	Not assigned/unknown	89	218	307	10	309	319	10	136	146

The upregulated, downregulated, and total DE genes for each treatment relative to control treatment (ambient CO_2_ and temperature) are shown.

### Biological Insight Into Metabolic Pathways of the Differentially Expressed Transcripts

**Figure 4:**
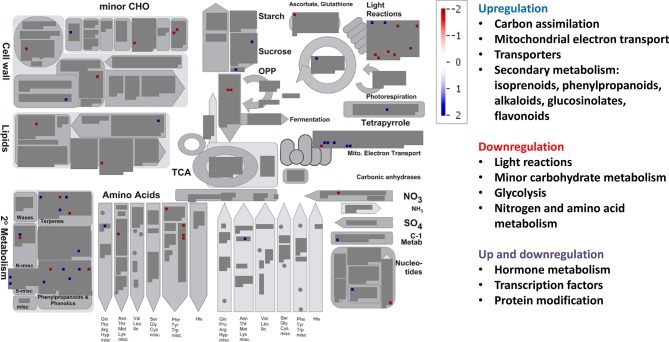
Significant changes in transcript levels associated with metabolic pathways under elevated [CO_2_] relative to the treatment under ambient [CO_2_] and temperature. White indicates no change, blue upregulation, and red downregulation as shown in the color key for a log fold-change scale. The main pathways affected associated with primary and secondary metabolism are highlighted.

**Figure 5:**
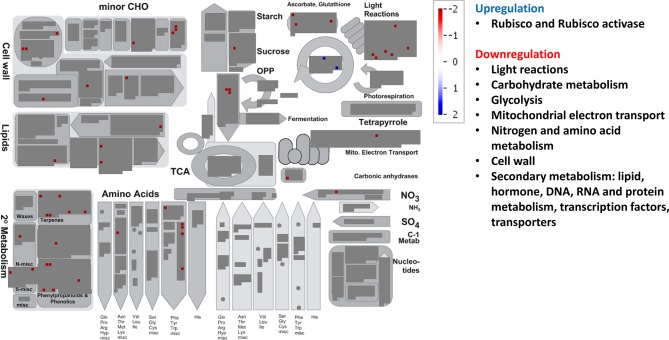
Significant changes in transcript levels associated with metabolic pathways under high temperature relative to the treatment under ambient [CO_2_] and temperature. White indicates no change, blue upregulation, and red downregulation as shown in the color key for a log fold-change scale. The main pathways affected associated with primary and secondary metabolism are highlighted.

**Figure 6:**
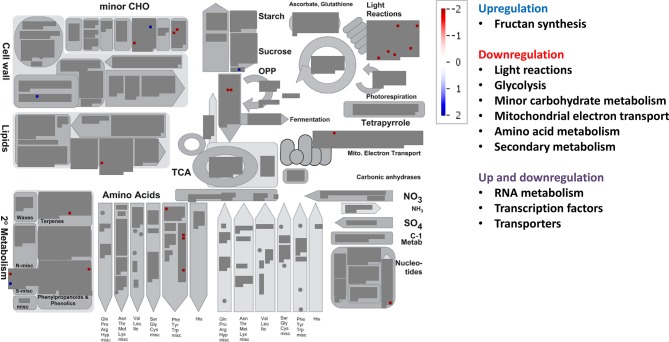
Significant changes in transcript levels associated with metabolic pathways under the combination of elevated [CO_2_] and high temperature relative to the treatment under ambient [CO_2_] and temperature. White indicates no change, blue upregulation, and red downregulation as shown in the color key for a log fold-change scale. The main pathways affected associated with primary and secondary metabolism are highlighted.

The MapMan BINs assigned to the transcripts allowed displaying gene expression results onto biological pathways using the MapMan application (**Figures 4**–**6**, **Supplementary Figure 4**). An overview of plant metabolism showed that EC increased the expression of several genes related to photosynthesis and carbohydrate metabolism relative to control treatment: two genes of light-harvesting complex II, CP12 (Calvin-Benson cycle), Mg protoporphyrin IX methyltransferase (tetrapyrrole synthesis), sucrose:fructan 6-fructosyltransferase (fructan synthesis), a vacuolar invertase, and a gene of raffinose synthase family protein (**Figure 4**, **Supplementary Figure 4**, **Supplementary Table 4**). However, seven genes from light harvesting (e.g. two photosystem II polypeptide subunits and several protein kinases) and three from minor carbohydrate metabolism (two involved in callose synthesis and one galactose mutarotase) were downregulated. EC also downregulated two genes of the cytosolic glyceraldehyde-3-phosphate dehydrogenase, involved in glycolysis pathway. EC strongly induced five of the six DE genes of mitochondrial electron transport (three cytochrome c oxidases and two ATP synthases). Although a gene involved in proline biosynthesis was upregulated, chiefly N metabolism was repressed by EC, as indicated by the downregulation of glutamate synthase, a gene of methionine synthesis, and four genes of the synthesis of aromatic amino acids (chorismate and tryptophan), and by the upregulation of one involved in the degradation of threonine. Most of the genes belonging to secondary metabolism were upregulated, including those involved in isoprenoid (e.g. carotenoids), phenylpropanoid (lignin), alkaloid, sulfur (glucosinolates), and flavonoid pathways. As for other plant functions, EC modified gene expression for hormone metabolism, inducing those related to gibberellins (DELLA protein) and jasmonates (12-oxophytodienoic acid reductase), and repressing those related to auxins, brassinosteroids, and ethylene (**Supplementary Table 4**). Eight of 11 genes related to RNA processing and transcription were downregulated, while the expression of 21 TFs from very diverse families was modified. Moreover, EC affected the expression of 46 genes involved in protein synthesis (mainly ribosomal proteins), posttranslational modification, and degradation (e.g. cysteine and serine proteases and ubiquitin). Regarding signaling functions, EC strongly reduced the transcript abundance of 53 out of 56 DE genes, including many receptor kinases (mainly leucine-rich repeat protein kinase family, DUF26 family of cysteine-rich receptor-like kinases, and cell wall–associated kinases) and other genes with unspecified functions. Transcript changes associated with transport revealed that, in general, EC induced the expression of transporters for amino acids (permease family protein), sulfate (transmembrane transporter), potassium (calcium-activated potassium channel), and peptides (proton-dependent oligopeptide transporters), as well as aquaporins (subfamily PIP) (**Supplementary Table 4**). EC repressed the expression of two porin genes and a plasma membrane ATP synthase, and modified the expression of genes encoding for several ABC and mitochondrial membrane transporters. Eighty-nine DE genes under EC were identified as stress-responsive genes, most of them downregulated relative to control treatment and lacking a good annotation. However, at variance with the other growth conditions, 12 stress-responsive genes were upregulated, mainly associated with heath shock proteins. The large enzyme families with more significant changes under EC were cytochrome P450 and oxidases (**Supplementary Table 4**).

Relative to control treatment, HT clearly repressed many metabolic pathways (**Figure 5**, **Supplementary Figure 4**). Thus, HT reduced the transcript abundance for genes involved in light reactions (diverse protein kinases associated with state transitions and a photosystem II polypeptide subunit), carbohydrate metabolism (galactose mutarotase and three enzymes involved in callose synthesis), glycolysis (3-phosphoglycerate kinase and two glyceraldehyde-3-phosphate dehydrogenases), mitochondrial electron transport (NADH dehydrogenase), cell wall (cellulose synthase, fasciclin-like arabinogalactan protein, leucine-rich repeat receptor protein kinase, glycoside hydrolase, and xyloglucan endotransglucosylase/hydrolase), lipid metabolism (several genes involved in fatty acid elongation, lipid transfer, and degradation), N and amino acid metabolisms (glutamate synthase and seven genes involved in the synthesis of methionine, chorismate, and tryptophan), and the glutathione–ascorbate cycle (**Figure 5**, **Supplementary Table 4**). Additionally, HT also repressed genes for secondary metabolism (related to non-mevalonate pathway, terpenoids, lignin, and other phenylpropanoids, alkaloids, glucosinolates, and flavonoids), a carbonic anhydrase, and an iron–sulfur enzyme related to NAD biosynthesis (quinolinate synthetase); and unexpectedly upregulated gene expression for ribulose-1,5-bisphosphate carboxylase oxygenase (Rubisco) large subunit and for Rubisco activase. All genes from hormone metabolism were also repressed by HT, such as those associated with abscisic acid, brassinosteroids, ethylene, gibberelins, and jasmonates. A total of 29 genes associated with RNA processing, transcription, binding, and regulation of transcription were downregulated, including TFs of the NAC, MYB, and WRKY families, auxin response factor, Constans-like zinc finger, and argonaute proteins, among others. Moreover, many genes involved in other regulation processes were downregulated, such as those related to DNA metabolism (DNA synthesis/chromatin structure and others with unspecified function), protein synthesis, posttranslational modification and degradation (similar to those altered under EC, but here all were downregulated), and signaling (leucine-rich repeat protein kinase family, DUF26 family of cysteine-rich receptor-like kinases, cell wall-associated kinases, LRK10 receptor-like protein kinases, and others associated with 14-3-3 proteins, phosphinositides, and light) (**Supplementary Table 4**). In transport category, we observed that HT repressed the expression of transporters for sugars (zinc induced facilitator), nitrate and ammonium (high affinity transporter members), peptides (proton-dependent oligopeptide transporters), as well as a plasma membrane ATP synthase, a cation efflux family protein, an ABC transporter, two porins, a cyclic nucleotide-gated ion channel, a mitochondrial substrate carrier family protein, and an aquaporin of TIP subfamily. Eighty-one DE genes corresponded to stress-responsive genes, all downregulated under HT relative to control treatment (**Supplementary Table 4**). Regarding changes in large enzyme families under HT, we highlight the downregulation of several members of cytochrome P450, UDP glucosyltransferases, oxidases, glutathione S-transferases, and GDSL-lipases, among others (**Supplementary Table 4**).

For the combined effects of EC and HT on gene expression, we observed lower number of total DE genes than under EC or HT (**Figure 3C**). EC × HT relative to control treatment decreased the expression of genes for light reactions (two photosystem II polypeptide subunits and some protein kinases associated with state transitions), minor carbohydrate metabolism (galactose mutarotase and two enzymes involved in callose synthesis), glycolysis (two glyceraldehyde 3-phosphate dehydrogenases), mitochondrial electron transport (NADPH:quinone oxidoreductase type 2), lipid degradation (glycerophosphodiester phosphodiesterase), amino acid metabolism (synthesis of chorismate and tryptophan), and secondary metabolism (related to terpenoids, phenylpropanoids, and synthesis of glucosinolates) (**Figure 6**, **Supplementary Figure 4**, **Supplementary Table 4**). In contrast, EC × HT upregulated the gene for sucrose:fructan 6-fructosyltransferase (fructan synthesis), aldose reductase (minor carbohydrate metabolism), xyloglucan endotransglucosylase (cell wall), fatty alcohol oxidase (lipid degradation), and another gene related to the degradation of glucosinolates. In the hormone metabolism, EC × HT only repressed two genes involved in the brassinosteroid signal transduction. The expression of genes for RNA metabolism were modified, with a general downregulation of those related to RNA transcription, binding, some TFs (basic helix-loop-helix and MYB families), an argonaute protein, and a DNA methyltransferases, while other TFs were upregulated (MADS box, MYB, and NAC families) (**Supplementary Table 4**). A few genes with unspecified function were downregulated in DNA metabolism, although some exonucleases and a helicase-like protein were overexpressed. Similar gene families related to protein synthesis, posttranslational modification and degradation, and signaling processes were downregulated under EC × HT relative to control as compared with HT, although the quantity was significantly lower. The transcript abundance of several transporters at the mitochondrial membrane and ABC transporters were altered (up and downregulated) under EC × HT, while those related with the transport of amino acids (amino acid–polyamine transporter), unspecified cations (organic cation/carnitine transporter), potassium (potassium ion transmembrane transporter), a plasma membrane ATP synthase, and a porin were downregulated. A total of 44 stress-responsive genes were downregulated, whereas a few genes of large enzyme families were modified by EC × HT: repression of two genes of cytochrome P450, three oxidases, and one alcohol dehydrogenase, and induction of two cytochrome P450 and two GDSL-lipases (**Supplementary Table 4**)

### Functional Enrichment Analysis Using Gene Ontology Terms

To complement functional categories based on MapMan annotations, we evaluated the GO terms associated with the DE genes. A total of 387, 376, and 213 GO terms were related to durum wheat responses to EC, HT, and the EC × HT interaction, respectively (**Supplementary Table 4**). Attending to the GO terms with the highest occurrence under the different environmental growth conditions, we found that the top five high frequency GO terms for molecular function, cellular component, and biological process were nearly the same for EC, HT, and EC × HT relative to control treatment. These GO terms were transferase activity, nucleotide binding, metal ion binding, oxidoreductase activity, and ATP binding for “molecular function”; cell, membrane, cytoplasm, integral component of membrane, and plasma membrane for “cellular component”; and cellular metabolic process, oxidation–reduction process, transmembrane transport, transcription, and regulation of transcription for “biological process.” Therefore, the main differences between the growth conditions were found in many GO terms with low frequency (**Supplementary Table 5**).

## Discussion

### Elevated CO_2_ Enhances Photosynthesis and Shoot Biomass and Leads to Changes in Central C-N Metabolism That Are Accompanied by Marked Changes in the Expression of Genes Involved in Photosynthesis, Amino Acid Metabolism, and Respiration

Photosynthesis has long been recognized as sensitive to environmental conditions. In our study, the exposure to EC enhanced flag leaf photosynthesis in good agreement with the well-documented stimulation of CO_2_ assimilation rate in response to CO_2_ enrichment in C_3_ plants (Long et al., 2004; Ainsworth and Rogers, 2007). Other widely observed response of plants to EC was a decrease in stomatal conductance (g_s_) (Long et al., 2004). In FACE experiments, the lower g_s_ at EC did not appear to be caused by a significant change in stomatal density (Estiarte et al., 1994). Therefore, it is likely that changes in stomatal aperture rather than density determine the response of g_s_ to EC (Ainsworth and Rogers, 2007). Gene expression analysis showed an increase in transcript abundance for genes annotated as S-type anion channel SLAH3 (SLAC1 homologue 3) and calcium-activated potassium channel, which are components of the guard cell signaling network for stomatal closure in response to CO_2_ (Laanemets et al., 2013) and could contribute to the g_s_ decrease. The greater shoot biomass accumulation was consistent with the increased photosynthetic rate at EC, as we have previously reported (Aranjuelo et al., 2011; Vicente et al., 2015a). Higher fructan and starch contents were also found in the flag leaves, which may be indicative of a sink limitation (Ainsworth et al., 2004). The fructan accumulation was associated with the induction of fructosyltransferases, as reported in rice (Fukayama et al., 2011) and wheat (Vicente et al., 2015a). The increase in photosynthesis caused by EC results in an increase in carbohydrate production, which may alter the C/N balance of wheat plants. Indeed, EC led to a decrease in the shoot N, on a weight and whole-organ basis, as well as the leaf N on a weight basis, suggesting that the plant N content of wheat plants decreased at EC (Taub and Wang, 2008; Aranjuelo et al., 2011), especially in leaf tissues (Seneweera et al., 2011; Gutiérrez et al., 2013; Vicente et al., 2015a). This decrease in N content could be associated with a limitation in N uptake or any other mechanism (del Pozo et al., 2007; Taub and Wang, 2008). Additionally, flag leaf NR activity was significantly decreased by EC. These results reflect that EC directly restrict leaf nitrate reduction, in agreement with other published works with wheat (Bloom et al., 2002; Bloom et al., 2010; Vicente et al., 2015a). However, higher (Vicente et al., 2016) or unaltered (Dier et al., 2017; Torralbo et al., 2019) NR activity in EC has been found. The decline with EC of foliar levels of most organic N compounds, such as Rubisco, amino acids, and soluble proteins, resembles previous findings (Bloom et al., 2002; Pérez et al., 2005; del Pozo et al., 2007; Gutiérrez et al., 2009a; Vicente et al., 2015a) and is consistent with an inhibition of N assimilation (Bloom et al., 2010). The fact that EC repressed the gene encoding glutamate synthase and genes linked to methionine and aromatic amino acid synthesis adds further support to a limitation in the assimilation of inorganic N into amino acids. A decline in foliar glutamine content, along with the increase in sucrose, could contribute to increase NR activation (Scheible et al., 1997; Morcuende et al., 1998). Interestingly, the approximately six-fold stronger induction of the proline synthesis enzyme delta-1-pyrroline-5-carboxylate synthetase by EC could lead to the accumulation of proline, which acts as a compatible osmolyte, protective agent for membranes and enzymes, scavenger of radicals, and/or transient storage form of organic N (Aswani et al., 2019).

The decrease in Rubisco amount, both in absolute terms and as a percentage of total soluble protein, could account for the lower Rubisco activity found in EC (Pérez et al., 2005), even though transcript levels for Rubisco large and small subunits were not altered. These findings indicate that gene transcription was not the only regulator of the enzyme. The maintenance of transcript abundance for Rubisco is consistent with our prior study in field-grown spring wheat at ear emergence (Pérez et al., 2005) and differs from the lower transcript levels found in durum wheat at anthesis (Vicente et al., 2015a), bread wheat (Nie et al., 1995), and rice (Fukayama et al., 2011; Seneweera et al., 2011). It therefore seems unlikely that sugar-mediated repression of Rubisco gene expression plays a dominant role in the loss in Rubisco protein found in our study (Moore et al., 1999), which presumably might be linked to a decrease in the leaf N status (Nakano et al., 1997; Pérez et al., 2005). The decrease in leaf glucose content could alleviate a possible carbohydrate repression of Rubisco gene expression (Moore et al., 1999). Although the foliar level of N compounds was lower under EC, the leaf chl content was not consistently decreased, possibly due to the increased transcripts for magnesium protoporphyrin IX methyltransferase (Alawady and Grimm, 2005). Likewise, two genes encoding light-harvesting complex (LHCII) were induced, whereas a set of genes encoding proteins involved in photosynthetic electron transport, including that encoding the photosystem II PsbR protein, were repressed (Vicente et al., 2015a), which may restrict the photosystem II functioning. Hence, it is tempting to speculate that a preferential decline of the photosystem proteins might be associated with a photo-oxidative damage, promoting proline accumulation to achieve redox homeostasis and counterbalance the adverse effect of the formation of reactive oxygen species (ROS).

In spite of the repression of a couple of genes encoding the glycolytic enzyme cytosolic glyceraldehyde-3-phosphate dehydrogenase (Vicente et al., 2015a; Vicente et al., 2016), dark respiration rates can be enhanced (Leakey et al., 2009; Markelz et al., 2014) owing to the carbohydrate buildup at EC. This is in line with the observation that genes encoding proteins linked to mitochondrial electron transport chain and transport on the mitochondrial membrane were strongly induced. These included different subunits of the cytochrome c oxidase and mitochondrial ATPase, along with a dicarboxylate transporter. Therefore, our observations are consistent with an enhancement in the leaf respiratory rates to provide C skeletons, energy, and reducing power for N assimilation and other metabolic pathways to ensure maintenance of plant metabolism and growth. Further research is needed to measure dark respiration rate and its relation with the respiratory enzyme activities and the level of primary metabolites.

### Elevated CO_2_ Also Leads to Widespread Changes in the Expression of Genes for Secondary Metabolism, Hormones, and Transcriptional Regulators

CO_2_ enrichment increased transcript levels for several genes linked to phenylpropanoid and isoprenoid biosynthetic pathways, e.g., a gene encoding 4-cuomarate CoA ligase 2 (4CL2), a key enzyme that provides the precursors for the synthesis of a large variety of secondary compounds, such as lignin, flavonoids, or phytoalexins (Stuible et al., 2000). Moreover, EC led to a decrease in transcript abundance for a cellulose synthase-like A protein that polymerizes the 1,4-β–linked backbone of mannans and glucomannans (Liepman and Cavalier, 2012), while increasing transcripts for a gene encoding an expansin-like A1 enzyme. This mediates the cell wall loosening that may contribute to cell wall expansion (Marowa et al., 2016). These data add support to the fact that the cell wall remodeling is vital for plant growth and leaf expansion at EC. Likewise, lignin accumulation may enhance the cell wall mechanical strength and protect against abiotic and biotic stresses (Liu et al., 2018). A large accumulation of secondary metabolites, including phenylpropanoids, alkaloids, glucosinolates, etc., has also been observed in plants exposed to elevated CO_2_ (Ghasemzadeh et al., 2010; Klaiber et al., 2013). Similarly, our data at transcriptional level resemble previous work with nitrate deficient tobacco plants that showed a marked decrease in the aromatic amino acid content, together with the induction of genes for secondary metabolism, the accumulation of products of the phenylpropanoid pathway, and the lignification of the stem (Fritz et al., 2006). Overall, the results point to a shift away from N-rich compounds towards increased levels of C-rich metabolites by regulating the distribution of photosynthetic C and assimilation of N to aromatic amino acids and phenylpropanoid biosynthesis, along with the closely related lignin synthesis (Cong et al., 2013). Our finding provides evidence that the impairment of primary metabolism induced by EC may have marked consequences for secondary metabolism, as reported previously in other plant species (Ainsworth et al., 2006).

EC also increased transcripts for a gene encoding a member of DELLA proteins, which promote the expression of downstream negative components of the gibberellin-signaling pathway providing a direct feedback mechanism for regulating gibberellin homeostasis (Zentella et al., 2007). This phytohormone mainly regulates cell elongation as well as other events as flowering and pollen maturation (Davière and Achard, 2013). The upregulation of genes encoding members of the 12-oxo-phytodienoic acid reductase protein family suggests an induction of jasmonic acid synthesis, at variance with the repression found in other studies (Zavala et al., 2008). Genes linked to auxin and ethylene metabolism were repressed (Zavala et al., 2008; Córdoba et al., 2017), as well as others related to brassinosteroids, although an induction has been reported by Jiang et al. (2012). Taken together, these results indicate a complex regulatory hormone metabolism towards growth modulation and adaptation to EC.

In agreement with previous findings by Ainsworth et al. (2006), the gene expression for members of different TF families was found to be influenced by EC, including bHLH, DOF, GATA, NAC, MADS-box, Homeobox, and WRKY, most of them being upregulated. It has recently been reported that WRKY family specifically responded to N deficiency in durum wheat (Curci et al., 2017), suggesting that it might contribute to N stress tolerance. Therefore, we might hypothesize that the transcriptional response of those TFs at EC could be associated to a decrease in the leaf N status.

Interestingly, EC increased transcripts for a set of genes encoding heat shock proteins, mainly HSP70, which act as molecular chaperones protecting proteins from aggregation, contributing to maintenance of protein homeostasis, translocation, and degradation (Wang et al., 2004). This result suggests that EC presumably promotes protein turnover, concurrent with the induction of several genes assigned to protein synthesis/degradation, and posttranslational modifications. Heat shock proteins have also been implicated in regulation of oxidative stress (Wang et al., 2004). In line with this observation, several genes encoding proteins involved in ROS detoxification and protection from oxidative damage were upregulated, as glutathione-S-transferase and β-carotene hydroxylase (You and Chan, 2015). A few orthologues to cytochrome P450 family, which catalyze the oxidation of different compounds in plants and are major players in detoxification of pesticides and other pollutants (Morant et al., 2003), were also upregulated. It is important to highlight that developmental processes are tightly regulated by redox states (Foyer and Noctor, 2011). The proline accumulation could also be related with the control of redox balance, as mentioned above. These findings provide evidence of the activation of a complex network for maintaining redox homeostasis.

Additionally, the transcript levels for several ABC transporters, which are involved in transport of phytohormones, peptides, sugars, alkaloids, inorganic acids, lipids, etc. (Kang et al., 2011), were increased. The upregulation of a gene encoding for oligopeptide transporter adds further support to the suggestion that protein turnover might occur in the flag leaf with subsequent transportation of N containing compounds, such as peptides. Consistent with this observation and the previous one, our data indicate that protein degradation in leaves is catalyzed by proteases through the proteasome ubiquitin system (Kurepa and Smalle, 2008). EC increased transcripts for a gene encoding a member of the UDP glucosyltransferase protein family. These mediate the transfer of sugars to a wide range of acceptor molecules, thus regulating certain properties such as their bioactivity, solubility, and transport within the cell and throughout the organism (Ross et al., 2001). A gene encoding a sulfate transporter was also induced. Taken together, these results reflect there were changes in cellular transport for maintenance of plant cellular homeostasis and growth in response to EC.

Finally, most of genes annotated as biotic or abiotic stress–related genes were strongly downregulated by EC. It may therefore be inferred that these plants were not subjected to significant stress conditions, in agreement with previous work of ourselves in barley (Córdoba et al., 2017) and of others in sugarcane (De Souza et al., 2008). A similar pattern of changes was also found in the expression of genes related to cellular functions as DNA synthesis and signaling (receptor kinases, sugar, and nutrients), in contrast with previous works (Ainsworth et al., 2006; Córdoba et al., 2017).

### High Temperatures Do Not Affect Photosynthesis and Nitrate Reductase Activity, But Decrease the Starch Content and Transcript Levels for Primary and Secondary Metabolism-Related Genes as Well as Those Required for Other Cellular Processes

In our experiment with durum wheat, 4 °C higher than AT did not inhibit photosynthesis, in agreement with our earlier reports in spring (Gutiérrez et al., 2009a) and durum wheat at later growth stages (Vicente et al., 2015a), and consequently had no significant impact on shoot biomass. However, the Rubisco protein content and the activity of the enzyme were decreased (Vu et al., 1997; Pérez et al., 2005), whereas the transcript levels for the Rubisco large subunit were increased. This result differs from the lower transcripts found not only for the Rubisco large subunit (Vicente et al., 2015a), but also for the small subunit in wheat (Pérez et al., 2005; Vicente et al., 2015a) and soybean (Vu et al., 1997). Interestingly, HT increased transcripts for a gene encoding a Rubisco activase, in spite of the usual decrease in Rubisco activation state in many plant species (Salvucci and Crafts-Brandner, 2004). This increase in our experiment could contribute to the maintenance of the Rubisco activation state. Furthermore, HT led to a decline in the starch and fructan contents that was accompanied by an increase in glucose, which may originate from the degradation of the former. These data presumably reflect the stimulation of C mobilization into multiple metabolic pathways and/or other organs for growth rate adjustment at higher temperatures.

Furthermore, there was a decreasing trend in NR activity at HT that was accompanied by similar changes in leaf and shoot N, on a weight and whole-organ basis, whereas the amino acid content remained unaltered and the transcript levels for several amino acid metabolism and nitrate and ammonium transporter genes were decreased. These findings show that HT does not restrict nitrate reduction and further amino acid biosynthesis at ear emergence, in contrast with the decrease reported at later growth stages (Vicente et al., 2015a). The fact that the leaf glucose content increased with HT could contribute to the maintenance of NR activity (Morcuende et al., 1998). Along with these changes, HT led to a general decline of transcripts involved in photosynthesis, cell wall synthesis, C metabolism, glycolysis, TCA cycle, and respiration, in agreement with our previous work (Vicente et al., 2015a). Such decrease was also observed in genes related to secondary metabolism, in contrast to the upregulation found at EC, reflecting a shift away from C-rich secondary metabolites as a mechanism to adjust C requirements according to cellular needs at HT.

Intriguingly, the majority of genes potentially involved in regulation, as those for hormone metabolism, protein kinases and phosphatases, receptor kinases, TFs, etc., were repressed (Bita and Gerats, 2013). The repression of genes involved in cell growth found in our experiments, such as histones and DNA polymerases, has also been reported in other studies (Sakata and Higashitani, 2008). It is worth nothing that moderately warm temperatures can trigger ROS generation and induce oxidative stress responses. Several studies have shown that moderate oxidative stress downregulates the expression of various genes including TFs (Morel and Barouki, 1999). Overall, the results presented here at the transcriptional level suggest that prolonged exposure to moderately warm temperatures have little effect on gene expression as compared to short-term heat stress. Further studies are required to assess the effects of extreme temperature events, rather than a continuous 4°C temperature increase, on photosynthesis, N content, biomass, and gene expression.

### The Transcriptional Response Induced by Elevated CO_2_ Combined With High Temperature Resembles That Reported for High Temperature Alone, Although It Was Partially Alleviated by Elevated CO_2_

In our study, wheat plants grown in EC and HT compared to those grown in AC and AT showed quite similar patterns of physiological and biochemical changes to plants exposed to EC as outlined above. Briefly, combined environmental factors led to a decrease in NR activity that was accompanied by a decline in leaf organic N compounds, including Rubisco protein, chl, and amino acids, whereas fructan content was increased in association with the upregulation of a gene encoding sucrose:fructan 6-fructosyltransferase, as in our prior study (Vicente et al., 2015a). The downregulation of genes linked to the shikimate pathway for the aromatic amino acids synthesis resembles previous findings in rice grown under low N (Xin et al., 2019) and is consistent with an inhibition of N assimilation and a decline in leaf N status.

Despite the repression of several genes involved in photosynthesis, C metabolism, glycolysis, and respiration under combined elevation of the factors studied, only a gene linked to phenylpropanoid and another linked to isoprenoid biosynthetic pathways were repressed, suggesting that possibly secondary metabolism is less sensitive to the effects of combined than separate elevation of these factors. Hence, it is possible that a proportion of excess C was diverted into secondary metabolism under HT and EC compared to HT alone. In addition, the transcript levels for a gene involved in glucosinolate degradation were increased. It is tempting to speculate that plants may catabolize glucosinolates to use the released sulfur to assist primary metabolism, such as protein synthesis in the leaf, allowing a readjustment to adverse conditions. Moreover, a gene encoding xyloglucan endotransglucosylase was induced, contrary to the repression observed at HT alone, which may contribute to cell wall loosening and elongation and the deposition of cellulose under combined factor elevation (Liu et al., 2007). Interestingly, most of the genes related to hormone metabolism remained unaltered compared to HT alone, except for the downregulation of two specific genes encoding brassinosteroids signaling proteins. This fact suggests that the hormonal response is translated into organ growth control (Wolters and Jurgens, 2009). Overall, the transcriptional response of plants to combined EC × HT was similar to, but weaker than, that reported for HT alone, indicating that it was partly attenuated by EC.

## Conclusions

The increasing threat of global warming on agricultural production worldwide requires dissecting the mechanisms that regulate plant responses not only to EC or HT, but also to the interaction of both factors, in order to develop stress-tolerant crops. The transcriptome sequencing is a powerful tool for the identification of relevant metabolic processes and underlying molecular mechanisms in the response of durum wheat to climate change. Confirming the hypothesis of the present study, the use of an integrated approach combining physiological and biochemical traits along with the transcriptome response has reported evidences that the inhibition of N assimilation triggered by EC led to a C/N imbalance in the flag leaf that was accompanied by an induction of secondary metabolism as a mechanism to divert excess C from central metabolism. The reprograming of the mitochondrial electron transport pathway was consistent with increased coupling of respiration to ATP production. It also provides novel information with respect to genes involved in the activation of cell expansion and growth, maintenance of protein homeostasis, and ROS detoxification and protection from oxidative damage in response to EC. In turn, the repression of genes associated to secondary metabolism driven by HT point to a shift away from C-rich secondary metabolites as a mechanism to adjust C requirements for growth under such environmental cue. The biochemical and transcriptional response under combined EC × HT provides new insights into the complex coordination of central metabolism with other secondary metabolic pathways involving plant hormones, transcriptional regulators, etc., in plant acclimation. These findings support that the combination of environmental factors imposes a specific metabolic demand compared to EC or HT alone, demonstrating the ability of plants to respond to complex environmental conditions that may occur under field conditions. This study has identified a comprehensive set of genes involved in modulation of durum wheat responses to future climate change.

## Data Availability Statement

The datasets generated for this study can be found in the European Nucleotide Archive (accession number PRJEB34302).

## Author Contributions

RM-C, PP, and RM conceived and designed the experimental setup, while RV, AB, BU, and RM designed the RNA sequencing analysis. RM-C, PP, EG, and RM conducted most of the physiological and biochemical analyses, while RV and AB carried out the transcriptome analyses. All authors assisted in analyses of data and discussed the results. RV and RM drafted the manuscript, and all authors revised and contributed to writing the manuscript.

## Funding

This research was supported by the Spanish National R&D&I Plan of the Ministry of Economy and Competitiveness [grants AGL2009-11987, AGL2013-41363-R (ERDF), and AGL2016-79589-R (ERDF)]. R. Vicente and E. Gutiérrez were the recipients of FPI fellowship from the Spanish Ministry of Economy and Competitiveness (BES-2010-031029) and I3P-European Fund fellowship, respectively.

## Conflict of Interest

The authors declare that the research was conducted in the absence of any commercial or financial relationships that could be construed as a potential conflict of interest.
