# Supplementary material for: De Novo Transcriptome Analysis of Durum Wheat Flag Leaves Provides New Insights Into the Regulatory Response to Elevated CO2 and High Temperature
Source: Front Plant Sci. 2019 Dec 6;10:1605. doi: 10.3389/fpls.2019.01605 (PMC6915051; doi:10.3389/fpls.2019.01605)
Supplement: Supplementary file 1 [file DataSheet_1.pdf]

Supplementary Material

Supplementary Table 1. RNA quantity and quality control after RNA extraction of the samples used for cDNA library construction and subsequent Illumina sequencing.

				NanoDrop ND-1000		Agilent 2100 Bioanalyzer
**Sample ID**	**[CO_2]**	**Temperature**	**RNA (µg/µl)**	**Ratio 260/280**	**Ratio 260/230**	**RIN[e]**
2003 AIH	Ambient	Ambient	1.09	2.08	2.23	9.1
2004 AIH	Ambient	Ambient	1.24	2.09	2.21	9.2
2005 AIH	Ambient	Ambient	1.32	2.13	2.25	9.0
2009 AFH	Ambient	Ambient + 4°C	1.20	2.10	2.17	8.9
2010 AFH	Ambient	Ambient + 4°C	1.31	2.07	2.18	8.5
2011 AFH	Ambient	Ambient + 4°C	1.19	2.06	2.18	8.8
2015 EIH	Elevated	Ambient	1.14	2.07	2.19	8.2
2016 EIH	Elevated	Ambient	1.21	2.07	2.17	8.3
2017 EIH	Elevated	Ambient	1.00	2.08	2.17	8.8
2021 EFH	Elevated	Ambient + 4°C	1.00	2.05	2.09	8.7
2022 EFH	Elevated	Ambient + 4°C	1.20	2.10	2.27	9.1
2023 EFH	Elevated	Ambient + 4°C	0.94	2.13	2.07	8.5

Supplementary Table 2. List of genes used for validation of RNA-Seq data by qRT-PCR. The primers used, which perfectly matched with the transcripts from the RNA-Seq dataset used for the comparison, can be found in Vicente et al. (2015).

**Acc. No.**	**Description**	**Symbol**
Ta2291	ADP-ribosylation factor	ADP-RF
AJ564996	Fructan 1-exohydrolase, *1-FEH w3* gene	1-FEH
AK332778	Pyruvate kinase	PK
AK333335	Fuctose-2,6-bisphosphatase/6-phosphofructo-2-kinase	F2KP
AY044844	Starch synthase, isoform IV	SSIV
BT009219	UTP-glucose-1-phosphate uridylyltransferase	UGPase
EF105343	ATP synthase B1 subunit, vacuolar	vATPase
GU563379	Putative pyruvate dehydrogenase E1 component α-subunit, mitochondrial	PDC

Supplementary Table 3. Sequencing results of RNA-Seq samples and reads mapped per sample using minimum kmer coverage of eight.

Sample ID	[CO_2]	Temperature	Illumina Mb	Illumina reads	Reads mapped	%
2003AIH1.f	Ambient	Ambient	4,712	47,122,153	81,924,697	89.07
2003AIH1.r	Ambient	Ambient	4,712	47,122,153		
2004AIH2.f	Ambient	Ambient	2,746	27,457,123	47,680,876	88.90
2004AIH2.r	Ambient	Ambient	2,746	27,457,123		
2005AIH3.f	Ambient	Ambient	3,273	32,734,387	55,020,114	85.92
2005AIH3.r	Ambient	Ambient	3,273	32,734,387		
2009AFH1.f	Ambient	Ambient + 4°C	3,107	31,070,633	54,142,146	89.27
2009AFH1.r	Ambient	Ambient + 4°C	3,107	31,070,633		
2010AFH2.f	Ambient	Ambient + 4°C	3,027	30,269,776	52,735,418	88.90
2010AFH2.r	Ambient	Ambient + 4°C	3,027	30,269,776		
2011AFH3.f	Ambient	Ambient + 4°C	2,598	25,980,321	45,761,051	89.82
2011AFH3.r	Ambient	Ambient + 4°C	2,598	25,980,321		
2015EIH1.f	Elevated	Ambient	3,359	33,588,078	58,316,553	89.00
2015EIH1.r	Elevated	Ambient	3,359	33,588,078		
2016EIH2.f	Elevated	Ambient	4,795	47,953,183	79,845,907	86.56
2016EIH2.r	Elevated	Ambient	4,795	47,953,183		
2017EIH3.f	Elevated	Ambient	4,518	45,179,137	78,845,189	89.10
2017EIH3.r	Elevated	Ambient	4,518	45,179,137		
2021EFH1.f	Elevated	Ambient + 4°C	3,136	31,361,528	54,561,563	89.04
2021EFH1.r	Elevated	Ambient + 4°C	3,136	31,361,528		
2022EFH2.f	Elevated	Ambient + 4°C	1,984	19,838,573	33,518,985	87.50
2022EFH2.r	Elevated	Ambient + 4°C	1,984	19,838,573		
2023EFH3.f	Elevated	Ambient + 4°C	2,268	22,681,863	38,281,976	87.26
2023EFH3.r	Elevated	Ambient + 4°C	2,268	22,681,863		

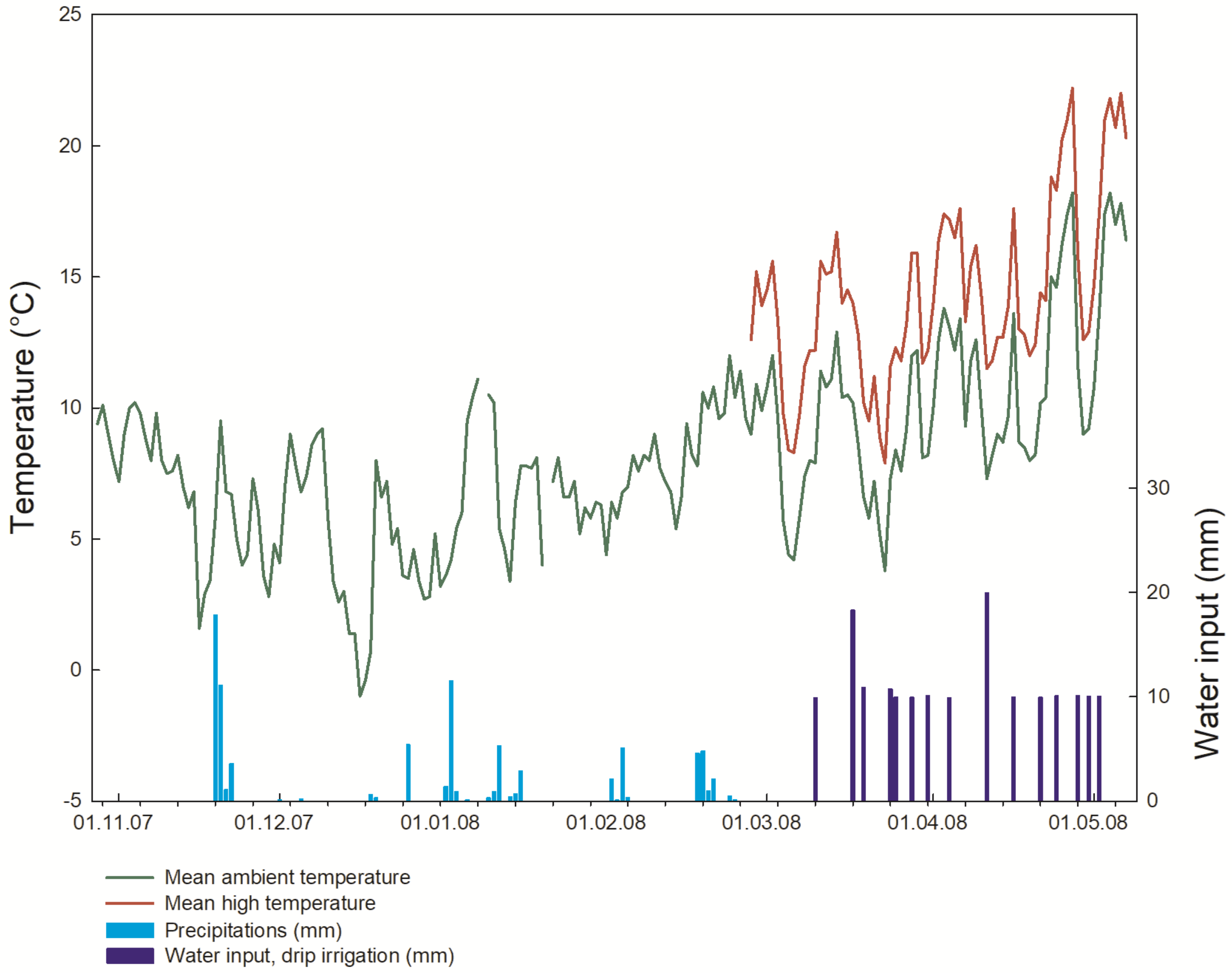

Supplementary Figure 1. Daily mean temperature in the field trial of durum wheat grown under ambient or high temperature. The water provided to the plants by precipitations or using the drip irrigation system is shown.

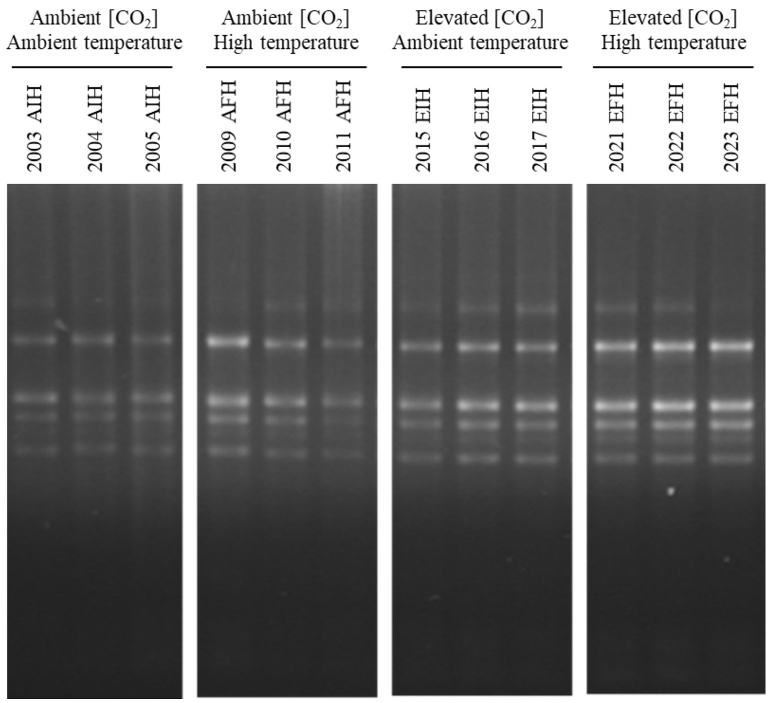

Supplementary Figure 2. RNA integrity of the samples used for Illumina sequencing after RNA isolation in 1.5% agarose gel. Samples 2003-2005: ambient [CO_2] and temperature; samples 2009-2011: ambient [CO_2] and 4 °C warmer temperatures; samples 2015-2017: elevated [CO_2] and ambient temperature; samples 2021-2023: elevated [CO_2] and 4 °C warmer temperatures.

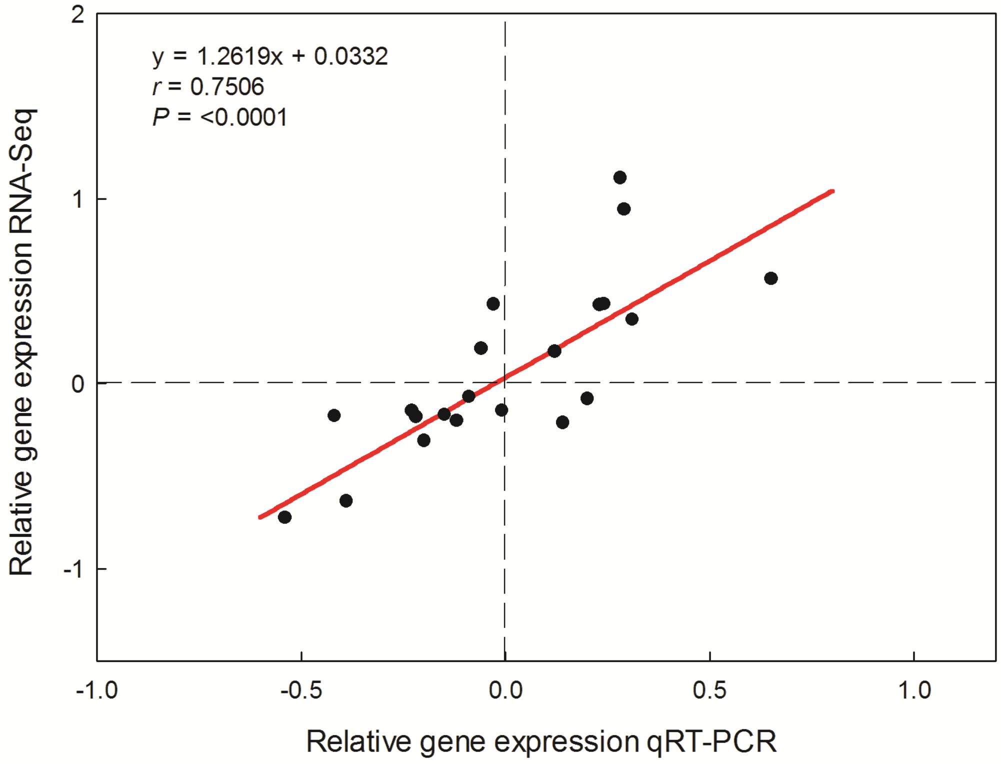

Supplementary Figure 3. Validation of RNA-Seq results by qRT-PCR. Relative gene expression of seven genes listed in Supplementary Table 1 by RNA-Seq and qRT-PCR analysis are compared for each treatment relative to treatment AC-AT using a scatterplot showing the correlation between both methods. The regression equation and coefficient (*r*) and *P* value are presented.

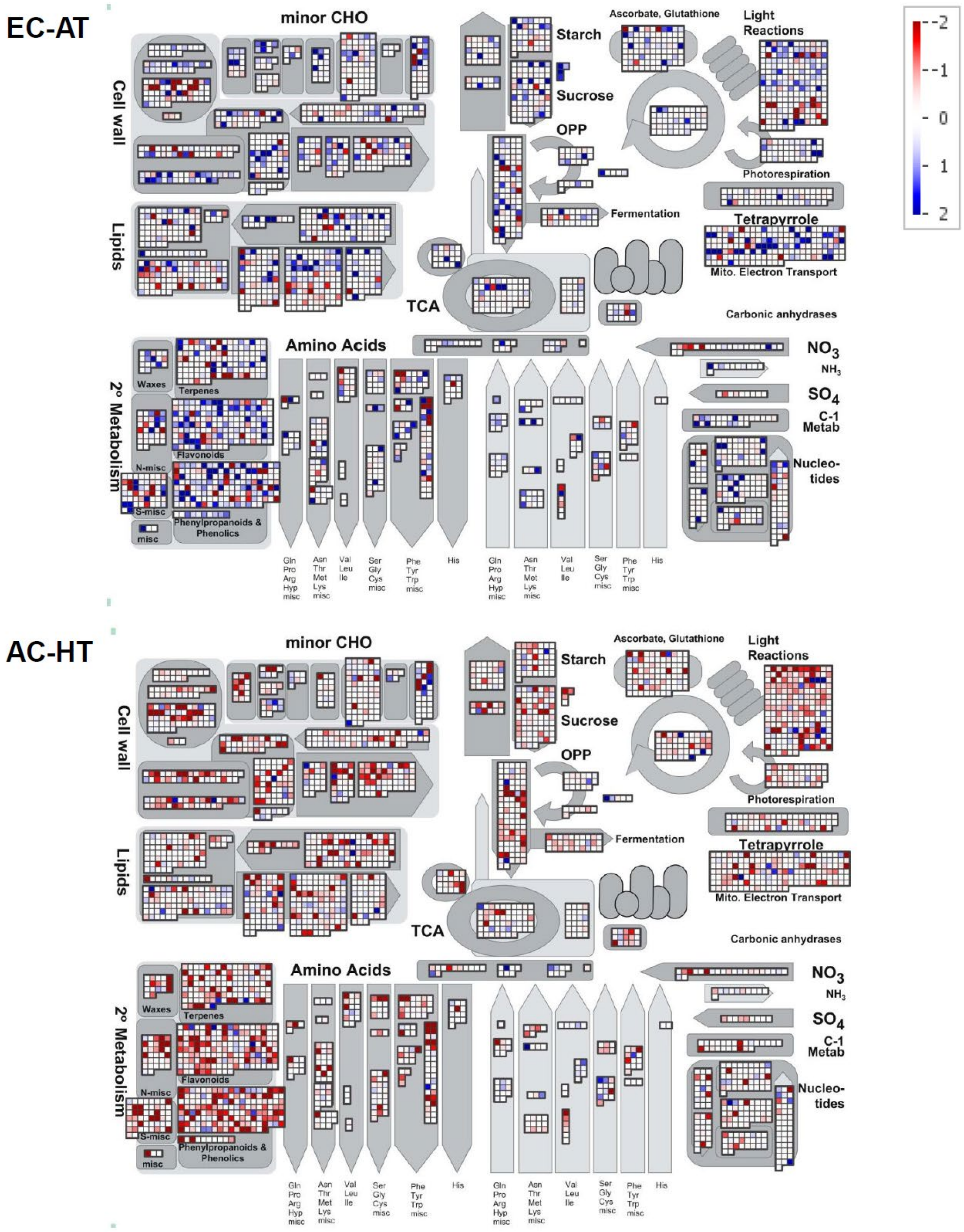

Supplementary Figure 4. Transcript changes associated with metabolic pathways under elevated [CO_2] (EC-AT), high temperature (AC-HT), or their combination (EC-HT) relative to the treatment under ambient [CO_2] and temperature (AC-AT). White indicates no change, blue upregulation and red downregulation as shown in the colour key for a log fold change scale.

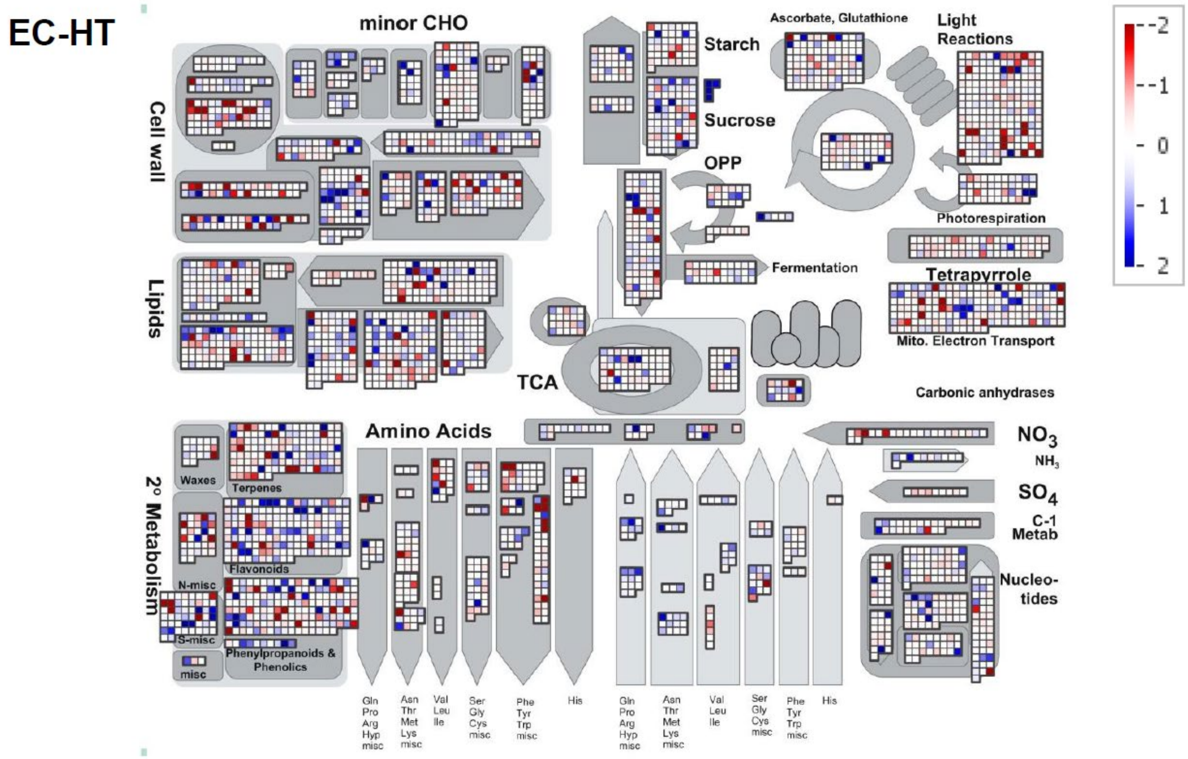

Supplementary Figure 4. Continued.
